# Increasing event‐driven HIV pre‐exposure prophylaxis use among gay, bisexual and other men who have sex with men in Australia: results from behavioural surveillance 2019–2023

**DOI:** 10.1002/jia2.26398

**Published:** 2024-11-24

**Authors:** Curtis Chan, Martin Holt, Anthony K. J. Smith, Timothy R. Broady, James MacGibbon, Limin Mao, Ben Wilcock, John Rule, Benjamin R. Bavinton

**Affiliations:** ^1^ Kirby Institute, UNSW Sydney Sydney New South Wales Australia; ^2^ Centre for Social Research in Health, UNSW Sydney Sydney New South Wales Australia; ^3^ Health Equity Matters Sydney New South Wales Australia; ^4^ National Association of People with HIV Australia Sydney New South Wales Australia

**Keywords:** HIV prevention, pre‐exposure prophylaxis, gay and bisexual men, men who have sex with men, event‐driven PrEP, on‐demand PrEP

## Abstract

**Introduction:**

HIV pre‐exposure prophylaxis (PrEP) has been publicly available since 2018 in Australia as a daily regimen. In 2019, clinical guidelines were updated to support guidance on event‐driven PrEP (ED‐PrEP) use. We assessed trends in the PrEP dosing regimen by comparing daily PrEP use to ED‐PrEP among cisgender gay, bisexual and other men who have sex with men (GBMSM).

**Methods:**

Data from repeated, cross‐sectional, national behavioural surveillance surveys were analysed from 2019 to 2023 among participants not living with HIV. Logistic regression models were conducted to assess trends and compared ED‐PrEP users to non‐PrEP users and daily PrEP.

**Results:**

Among 38,880 participants, overall PrEP use with any regimen increased from 27.6% in 2019 to 42.7% in 2023 (OR = 1.16, 95% CI = 1.15−1.18, *p* < 0.001). Among 12,922 participants who reported PrEP use in the last 6 months, the proportion reporting ED‐PrEP use increased from 7.6% in 2019 to 27.8% in 2023 (OR = 1.41, 95% CI = 1.37−1.46, *p* < 0.001) with those who reported daily PrEP decreasing from 92.4% to 63.3% (OR = 0.64, 95% CI = 0.62−0.66, *p* < 0.001). In a cross‐sectional sub‐sample in 2022–2023 (*n* = 8840), compared to ED‐PrEP users, non‐PrEP users were less likely to have received three or more HIV tests in the last 12 months (aRRR = 0.26, 95% CI = 0.22−0.31, *p* < 0.001), have 2−10 male sexual partners in the last 6 months (aRRR = 0.24, 95% CI = 0.14−0.41, *p* < 0.001) or 11 or more (aRRR = 0.26, 95% CI = 0.15−0.45, *p* < 0.001) compared to none, or to report condomless anal intercourse with casual partners (aRRR = 0.38, 95% CI = 0.32−0.46, *p* < 0.001). Compared to ED‐PrEP users, daily PrEP users were more likely to have received three of more HIV tests in the last year (aRRR = 3.73, 95% CI = 3.15−4.40, *p* < 0.001) and less likely to be born overseas and lived in Australia for less than 5 years compared to being born in Australia (aRRR = 0.64, 95% CI = 0.49−0.83, *p* = 0.001).

**Conclusions:**

While daily PrEP remains the most common PrEP dosing regimen among GBMSM in Australia, there has been a steep increase in the proportion of PrEP users who are taking ED‐PrEP. Monitoring of PrEP use should continue to adapt to new dosing methods and future PrEP options. As ED‐PrEP use increases, further work is needed to ensure those taking ED‐PrEP are taking it effectively to prevent HIV.

## 1 INTRODUCTION

HIV pre‐exposure prophylaxis (PrEP) has been shown to be an effective way of preventing HIV. Event‐driven PrEP (ED‐PrEP), also known as “on‐demand PrEP” or “2‐1‐1 PrEP,” involves taking a loading dose of two pills 2–24 hours before sex, then one pill per day until 2 days have elapsed since the last sexual contact [1, 2]. ED‐PrEP has been recognized in international PrEP guidelines for at least 5 years and is currently recommended only for cisgender gay, bisexual and other men who have sex with men (GBMSM) [1, 3–5]. Cohort studies from France [6] and West Africa [7] have found high proportions of participants choosing ED‐PrEP over daily, at 49.5% and 72.1%, respectively. Data from PrEP users in The Netherlands and Belgium found 25.9% initially chose ED‐PrEP over daily PrEP [8]. Cross‐sectional studies from Germany and Taiwan found 26.9% and 56% of PrEP users choosing ED‐PrEP, respectively [9, 10].

There is some evidence that awareness and use of ED‐PrEP has been growing in Australia. In 2020, 68.4% of previous participants from a PrEP demonstration project in New South Wales had heard of ED‐PrEP, but only 12.5% knew how to take it per the 2‐1‐1 method [11]. In 2021, a cross‐sectional survey of GBMSM (both PrEP and non‐PrEP users) found 68.3% heard of it, and 43.7% knew the components of the 2‐1‐1 method [12]. The first iterations of national PrEP prescribing guidelines in Australia did not include ED‐PrEP until 2019 [4, 13, 14]. As the cost of PrEP is a known barrier to PrEP use [15, 16, 17], ED‐PrEP is a potentially more cost‐effective option for those who want to purchase PrEP less frequently while remaining protected during periods of risk [18, 19]. While these studies indicate greater awareness of ED‐PrEP, there has yet to be an analysis of trends in ED‐PrEP use among Australian GBMSM.

This study aimed to determine trends in ED‐PrEP use among Australian GBMSM using HIV behavioural surveillance data, and to determine factors associated with ED‐PrEP use. This analysis helps understand who is using ED‐PrEP and how they may be supported to use it effectively, as well as identifying characteristics of potential users who may benefit from ED‐PrEP but are not currently using this method including daily PrEP users or those who are not yet taking PrEP.

## 2 METHODS

### 2.1 Participants

The methods of the Gay Community Periodic Surveys have been described elsewhere [20]. Briefly, the Gay Community Periodic Surveys are repeated national cross‐sectional surveys of GBMSM in Australia. Participants are recruited face‐to‐face during Pride festival seasons at events, venues and clinics, and further supplemented by online recruitment [20]. Eligible participants were Australian residents who were men (trans‐inclusive), aged 18 or over for face‐to‐face recruitment or 16 or over for online recruitment, and self‐identified as GBMSM. Verbal consent was obtained from participants who completed the survey in‐person, and participants completing the survey online provided electronic consent. Ethical approval was received from the University of New South Wales Human Research Ethics Committee (HC180903), and the research ethics committees of the community organizations ACON and Thorne Harbour Health.

### 2.2 Measures

In 2019 and 2020, PrEP use was measured with the question, “In the last 6 months, did you take PrEP to protect yourself from HIV?” (“No,” “Yes, I took it daily/most days” or “Yes, I took it around the time of sex (but not daily)”). Participants who ticked either of the last two options were considered to have taken PrEP in the last 6 months. From 2021 onwards, PrEP use was assessed with two questions: “In the last 6 months, did you take PrEP to protect yourself from HIV?” (“Yes” or “No”) and “If you took PrEP in the last 6 months, how did you take it?” (“Daily/most days,” “Around the time of sex (on‐demand, 2‐1‐1),” “Daily for a limited period of time, e.g. a month,” “Another way” or “Did not take PrEP”). Responses from 2019 and 2020 were recoded to the corresponding response options in the 2021–2023 survey.

Demographic measures included age, sexual identity, current employment status, highest level of education (dichotomized as having a university education vs. lower education level), country of birth and recency of arrival (if born overseas), and relationship status. Each participant's residential postcode was categorized based on the estimated proportion of gay‐identified men in that postcode using an established method [21]. These were categorized as “<5% gay,” “5 to <10% gay” and “≥10% gay” residents. Gay social engagement was measured by asking about the number of friends who were gay men, and the free time spent with gay men [22]. The number of HIV tests participants reported in the last 12 months were dichotomized to “two or fewer” and “three or more.” Participants self‐reported if they had been diagnosed any sexually transmitted infection (STI) within the last year. Participants reported the number of male sexual partners they had in the last 6 months and were categorized into “None,” “1,” “2−10” or “11 or more.” Casual sex in the last 6 months was dichotomized into any condomless anal intercourse with casual partners (CLAIC) versus not, and sex with regular male partners in the last 6 months was dichotomized into any condomless anal intercourse with regular partners (CLAIR) versus not.

### 2.3 Analyses

Analyses were restricted to participants who were not living with HIV. Overall trends in PrEP use (daily, ED‐PrEP and both) were assessed using logistic regression. Sensitivity analyses were conducted by restricting the trend analyses to 2019–2021 and 2021–2023 to account for the change in how the PrEP questions were asked after 2021. A cross‐sectional analysis of the last available round from each state/territory from either 2022 (South Australia) or 2023 (New South Wales, Victoria, Queensland, Western Australia and Tasmania) was conducted to compare characteristics of ED‐PrEP users to non‐PrEP users and daily PrEP users using multinomial logistic regression with ED‐PrEP users as the reference group, excluding those who reported taking PrEP in another way. Variables significant at the bivariable level were entered into a multivariable multinomial logistic regression model. Unadjusted odds ratios (OR), unadjusted relative risk ratios (RRR) and adjusted relative risk ratios (aRRR), 95% confidence intervals and *p*‐values are reported. Statistical significance was set at α = 0.05.

## 3 RESULTS

Between 2019 and 2023, 42,044 participants were recruited with 22,840 (54.3%) in face‐to‐face venues and 19,204 (45.7%) from online recruitment. The response rate was 70.6% among face‐to‐face participants and the participation rate was 76.7% among online participants. Participants living with HIV (*n* = 3164, 7.6%) were excluded from further analysis. Of the remaining 38,880 participants, the mean age was 37.6 years (SD = 11.5) and 11,346 (88.8%) identified as gay, with another 887 (6.9%) identifying as bisexual, and 509 (4.0%) a different sexual identity. There were 4201 (33.0%) participants who were born in a country outside of Australia, with 2931 (70.1%) of these having lived in Australia for over 5 years.

**Figure 1:**
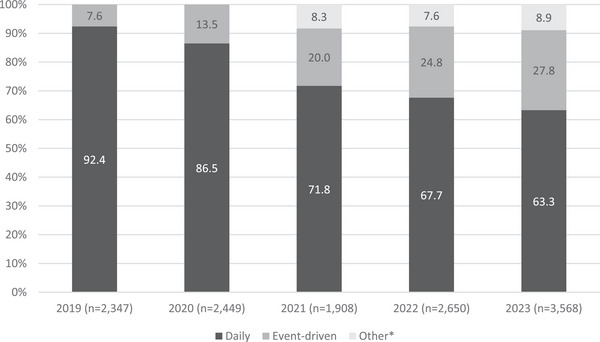
Pre‐exposure prophylaxis (PrEP) use regimen among PrEP users, 2019–2023 (*n* = 12,922). *“Other” option introduced in 2021.

Of the included 38,880 participants, 12,922 (32.9%) reported PrEP use in the last 6 months. The proportion of participants who reported PrEP use in the last 6 months increased from 27.6% in 2019 to 42.7% in 2023 (OR = 1.16, 95% CI = 1.15−1.18, *p* < 0.001), with statistically significant upward trends for both 2019–2021 and 2021–2023 (Table 1). Daily PrEP users as a proportion of the total sample remained similar from 25.5% in 2019 to 27.0% in 2023 (OR = 1.00, 95% CI = 0.98−1.01, *p* = 0.706). At the same time, ED‐PrEP use increased from 2.1% in 2019 to 11.9% in 2023 (OR = 1.52, 95% CI = 1.48−1.57, *p* < 0.001; Table 1). Among the 12,922 participants who reported taking PrEP, the proportion who reported daily PrEP decreased from 92.4% in 2019 to 63.3% in 2023 (OR = 0.64, 95% CI = 0.62−0.66, *p* < 0.001). ED‐PrEP increased from 7.6% in 2019 to 27.8% in 2023 (OR = 1.41, 95% CI = 1.37−1.46, *p* < 0.001; Figure 1).

**Table 1:** Overall pre‐exposure prophylaxis (PrEP) use and regimen, and trend analyses among the whole sample (*n* = 38,880)

	2019	2020	2021	2022	2023	Odds ratio (95% CI) 2019–2021	Odds ratio (95% CI) 2021–2023	Odds ratio (95% CI) 2019–2023
Any PrEP use in the last 6 months	2347 (27.6)	2449 (31.2)	1908 (29.8)	2650 (34.3)	3568 (42.7)	1.06 (1.02−1.09)^**^	1.31 (1.26−1.35)^***^	1.16 (1.15−1.18)^***^
Daily PrEP	2169 (25.5)	2119 (27.0)	1369 (21.4)	1793 (23.2)	2257 (27.0)	0.90 (0.87−0.94)^***^	1.17 (1.13−1.22)^***^	1.00 (0.98−1.01)
Event‐driven‐PrEP	178 (2.1)	330 (4.2)	381 (5.9)	657 (8.5)	992 (11.9)	1.68 (1.54−1.83)^***^	1.46 (1.37−1.55)^***^	1.52 (1.48−1.57)^***^
Other	−	−	158 (2.5)	200 (2.6)	319 (3.8)			
No PrEP use in the last 6 months	6161 (72.4)	5411 (68.8)	4505 (70.3)	5084 (65.7)	4797 (57.4)	−	−	−
Total	8508	7860	6413	7734	8365			

** *p* < 0.01.

*** *p* < 0.001.

In the cross‐sectional sample in 2022–2023 (*n* = 9177), there were 337 participants who reported taking PrEP another way and were excluded from further analysis. Among the remaining 8840 participants, 2392 (26.1%) reported taking PrEP daily, 1078 (11.8%) reported taking ED‐PrEP and 5370 (58.5%) reported no PrEP use in the last 6 months. Bivariable results are reported in Table 2.

**Table 2:** Comparing event‐driven pre‐exposure prophylaxis (ED‐PrEP) users to daily PrEP users and non‐PrEP users in cross‐sectional sample in 2022–2023 (*n* = 8840)

				ED‐PrEP versus Daily PrEP		ED‐PrEP versus No PrEP use	
	ED‐PrEP	Daily PrEP	No PrEP use	Relative risk ratio (95% CI)	Adjusted relative risk ratio (95% CI)	Relative risk ratio (95% CI)	Adjusted relative risk ratio (95% CI)
Age (mean/standard deviation)	38.6 (12.6)	38.1 (11.3)	38.9 (14.5)	1.00 (0.99−1.00)		1.00 (1.00−1.01)	
Sexual identity							
Gay	935 (86.9)	2092 (87.7)	3902 (73.2)	REF	REF	REF	REF
Bisexual	88 (8.2)	180 (7.5)	871 (16.3)	0.91 (0.70−1.19)	1.09 (0.82−1.45)	2.37 (1.88−2.99)^***^	1.88 (1.45−2.44)^***^
Other	53 (4.9)	114 (4.8)	557 (10.5)	0.96 (0.69−1.34)	0.89 (0.62−1.26)	2.52 (1.88−3.37)^***^	1.73 (1.23−2.44)^**^
Full time employment	801 (74.3)	1871 (78.2)	3513 (65.4)	1.24 (1.05−1.47)^*^	1.15 (0.96−1.38)	0.65 (0.56−0.76)^***^	0.87 (0.73−1.04)
University education	765 (71.1)	1593 (66.7)	2991 (55.9)	0.81 (0.70−0.95)^*^	0.76 (0.64−0.91)^**^	0.52 (0.45−0.59)^***^	0.56 (0.48−0.67)^***^
Migration experience							
Born in Australia	655 (60.9)	1566 (65.7)	3656 (68.4)	REF	REF	REF	REF
Born overseas—lived in Australia more than 5 years	301 (28.0)	639 (26.8)	1252 (23.4)	0.89 (0.75−1.05)	0.88 (0.74−1.05)	0.74 (0.64−0.87)^***^	0.87 (0.73−1.04)
Born overseas—lived in Australia 5 years or less	119 (11.1)	180 (7.5)	437 (8.2)	0.64 (0.49−0.81)^***^	0.64 (0.49−0.83)^**^	0.66 (0.53−0.82)^***^	0.85 (0.66−1.11)
Postcode category by estimated proportion of gay male population							
<5% gay	611 (58.0)	1242 (52.8)	3641 (69.9)	REF	REF	REF	REF
5 to <10% gay	238 (22.6)	510 (21.7)	922 (17.7)	1.05 (0.88−1.36)	0.99 (0.81−1.20)	0.64 (0.55−0.77)^***^	0.89 (0.73−1.09)
≥10% gay	204 (19.4)	599 (25.5)	644 (12.4)	1.44 (1.20−1.73)^***^	1.33 (1.09−1.64)^**^	0.53 (0.44−0.63)^***^	0.75 (0.60−0.94)^*^
Gay social engagement (mean/standard deviation)	5.5 (1.4)	5.6 (1.4)	4.7 (1.6)	1.08 (1.03−1.14)^**^	1.01 (0.95−1.06)	0.74 (0.71−0.77)^***^	0.89 (0.84−0.94)^***^
Relationship status							
Open	404 (38.0)	764 (32.4)	746 (15.1)	REF	REF	REF	REF
Monogamous	54 (5.1)	163 (6.9)	1568 (31.7)	1.55 (1.13−2.13)^**^	2.53 (1.73−3.70)^***^	14.59 (11.00−19.37) ^***^	4.14 (2.94−5.85)^***^
Casual only	554 (52.2)	1346 (57.0)	1359 (27.5)	1.28 (1.10−1.50)^**^	1.31 (1.10−1.55)^**^	1.32 (1.13−1.55)^***^	1.13 (0.94−1.35)
No sex with men	50 (4.7)	88 (3.7)	1273 (25.7)	0.92 (0.64−1.33)	1.37 (0.85−2.21)	13.45 (9.90−18.28)^***^	1.45 (0.96−2.20)
Received three or more HIV tests in the last 12 months	514 (47.8)	1886 (79.2)	479 (9.0)	4.13 (3.54−4.83)^***^	3.73 (3.15−4.40)^***^	0.10 (0.09−0.13)^***^	0.26 (0.22−0.31)^***^
Any STI diagnosis in the last 12 months	347 (32.2)	1091 (45.6)	446 (8.3)	1.77 (1.52−2.05)^***^	1.14 (0.96−1.35)	0.19 (0.16−0.22)^***^	0.58 (0.48−0.70)^***^
Number of male partners in the last 6 months							
None	24 (2.2)	42 (1.8)	1130 (21.8)	REF	REF	REF	REF
1	87 (8.1)	156 (6.5)	2038 (39.3)	1.00 (0.57−1.76)	0.68 (0.35−1.34)	0.49 (0.31−0.77)^**^	0.59 (0.34−1.03)
2−10	683 (63.8)	1062 (44.5)	1666 (32.1)	0.87 (0.52−1.45)	0.57 (0.30−1.08)	0.05 (0.03−0.08)^***^	0.24 (0.14−0.41)^***^
11 or more	277 (25.9)	1124 (47.1)	355 (6.8)	2.27 (1.36−3.81)^**^	1.16 (0.60−2.23)	0.03 (0.02−0.04)^***^	0.26 (0.15−0.45)^***^
Condomless anal intercourse with casual partners in the last 6 months	751 (69.7)	1832 (76.6)	1098 (20.4)	1.42 (1.21−1.67)^***^	1.04 (0.85−1.26)	0.11 (0.10−0.13)^***^	0.38 (0.32−0.46)^***^
Condomless anal intercourse with regular partners in the last 6 months	708 (65.7)	1665 (69.6)	2362 (44.0)	1.20 (1.03−1.39)^*^	1.08 (0.91−1.29)	0.41 (0.36−0.47)^***^	0.64 (0.54−0.76)^***^
Total	1078	2392	5370				

* *p* < 0.05.

** *p* < 0.01.

*** *p* < 0.001.

In the multivariable analysis, compared to ED‐PrEP users, daily PrEP users were more likely live in a postcode with ≥10% gay men (aRRR = 1.33, 95% CI = 1.09−1.64, *p* = 0.006), be in a monogamous relationship (aRRR = 2.53, 95% CI = 1.63−3.70, *p* < 0.001) or have casual partners only (aRRR = 1.31, 95% CI = 1.10−1.55, *p* = 0.002), or report three of more HIV tests in the last year (aRRR = 3.73, 95% CI = 3.15−4.40, *p* < 0.001). Daily PrEP users were less likely to have a university education (aRRR = 0.76, 95% CI = 0.91, *p* = 0.002) or to be born overseas and have lived in Australia for less than 5 years compare to being born in Australia (aRRR = 0.64, 95% CI = 0.49−0.83, *p* = 0.001; Table 2).

In the multivariable analysis, compared to ED‐PrEP users, those who did not take PrEP were more likely to identify as bisexual (aRRR = 1.88, 95% CI = 1.45−2.44, *p<*0.001) or another sexual identity (aRRR = 1.73, 95% CI = 1.23−2.44, *p* = 0.002) or be in a monogamous relationship compared to an open relationship (aRRR = 4.13, 95% CI = 2.94−5.85, *p* < 0.001), and were less likely to have a university education (aRRR = 0.56, 95% CI = 0.48−0.67, *p* < 0.001), live in a postcode with ≥10% gay men compared to <5% gay men (aRRR = 0.75, 95% CI = 0.60−0.94, *p* = 0.011), be socially engaged with gay men (aRRR = 0.89, 95% CI = 0.84−0.94, *p* < 0.001), to have received three or more HIV tests in the last 12 months (aRRR = 0.26, 95% CI = 0.22−0.31, *p* < 0.001), received any STI diagnosis in the previous 12 months (aRRR = 0.58, 95% CI = 0.48−0.70, *p* < 0.001), have 2−10 male sexual partners in the last 6 months (aRRR = 0.24, 95% CI = 0.14−0.41, *p* < 0.001) or 11 or more (aRRR = 0.26, 95% CI = 0.15−0.45, *p* < 0.001) compared to none, or report CLAIC (aRRR = 0.38, 95% CI = 0.32−0.46, *p* < 0.001) or CLAIR (aRRR = 0.64, 95% CI = 0.54−0.76, *p* < 0.001) in the last 6 months (Table 2).

## 4 DISCUSSION

In our large sample of Australian GBMSM, overall PrEP use using either daily or event‐driven dosing increased substantially between 2019 and 2023. The proportion of participants using daily PrEP decreased from nearly all PrEP users in 2019 to two‐thirds of PrEP users in 2023. The proportion of ED‐PrEP users more than tripled, accounting for over a quarter of PrEP users in 2023 with increasing ED‐PrEP contributing to the overall increase in PrEP use. In the cross‐sectional analysis, ED‐PrEP use was associated with university education, being a recent migrant, area of residence, relationship status and HIV testing frequency.

There are several possible influences that may have facilitated the overall increase in ED‐PrEP uptake we observed. There is increased awareness of PrEP and event‐driven dosing among GBMSM [12, 23]. While we found a steep increase in ED‐PrEP use, other countries have demonstrated higher use of ED‐PrEP, possibly reflecting greater support from policy and clinical guidelines at earlier points than in Australia [8, 10, 24, 25]. During the study period, COVID‐19 caused disruptions to sexual behaviour and PrEP use, potentially prompting PrEP users to consider alternatives to daily dosing [20, 26, 27]. The adoption into clinical guidelines, awareness campaigns and reduced sexual activity could have contributed to the steep increase in ED‐PrEP use we observed.

We identified several factors associated with ED‐PrEP use compared to daily PrEP users and non‐PrEP users. Recently arrived migrant GBMSM were more likely to take ED‐PrEP than daily PrEP. Recent migrants are less likely to have access to subsidized medication in Australia, and ED‐PrEP is more affordable than daily dosing [14, 17, 28–30]. Our results also showed that ED‐PrEP users are more health service engaged than non‐users, but significantly less engaged than daily PrEP users in HIV testing. ED‐PrEP users were more sexually active than non‐PrEP users, and engaged in similar levels of sexual activity as daily PrEP users. Retaining ED‐PrEP users in care for regular testing may become an ongoing challenge as previous evidence shows overall decreasing levels of frequent testing among GBMSM, including among PrEP users [31]. Conversely, those who lived in areas with an estimated higher density of gay men were more likely to take daily PrEP rather than ED‐PrEP. As early PrEP uptake was faster in these areas [32, 33], early adopters of PrEP may have become accustomed to daily PrEP and still prefer it to ED‐PrEP.

Further promoting ED‐PrEP could facilitate greater PrEP coverage but work is needed to support effective use of this method. Previous work on ED‐PrEP knowledge has shown a large proportion of GBMSM could not correctly recall the 2‐1‐1 method, including those who reported that they were taking ED‐PrEP [11, 12, 34, 35]. Promoting ED‐PrEP could encourage potential users to keep PrEP pills readily available even in periods without sexual activity and to restart PrEP when sex is anticipated.

There are some limitations to this study. Our study did not capture whether participants switched between daily and ED‐PrEP in the last 6 months. Prior to 2021, the survey questionnaire did not directly mention ED‐PrEP, but asked about PrEP use “around the time of sex.” Some participants who we considered ED‐PrEP users may not have been referring to the 2‐1‐1 method specifically in those earlier years. The survey did not assess awareness or knowledge of ED‐PrEP among participants, nor adherence to PrEP for daily or ED‐PrEP users.

## 5 CONCLUSIONS

Our findings indicate that among GBMSM taking PrEP in Australia, daily PrEP remains the most well‐established dosing regimen with ED‐PrEP increasing rapidly. Promoting ED‐PrEP to those who may suit this regimen due to concerns about cost, such as recent migrants, should be considered. Greater awareness of different PrEP dosing options could help improve retention and lower perceived barriers to initiation. The benefits of ED‐PrEP, including identifying more populations who would be best suited to its use, should be considered and continued monitoring of its use is recommended.

## COMPETING INTERESTS

In the last 3 years, BRB has received payment or honoraria from Gilead Sciences, ViiV Healthcare and FHI 360, and received unrestricted research grants from Gilead Sciences and ViiV Healthcare. TRB has received payment from Gilead to provide expert feedback at an online workshopping event. For the remaining authors, no conflicts were declared.

## AUTHORS’ CONTRIBUTIONS

CC, BRB and MH contributed to the conceptualization of the topic. CC and TRB contributed to the data curation. CC analysed the data and drafted the paper. Funding was acquired by BRB, LM, TRB and MH. All authors read, commented on and approved the final manuscript.

## FUNDING

The study was funded by the National Health and Medical Research Council Partnership Project (GNT2002625), the Australian Government Department of Health and state/territory health departments.

## Data Availability

Deidentified data and syntax used in this analysis will be shared to researchers after a request to the corresponding author.
